# Preparation and Tribological Properties of Carbon-Coated WS_2_ Nanosheets

**DOI:** 10.3390/ma12172835

**Published:** 2019-09-03

**Authors:** Zheng Li, Fanshan Meng, Haohao Ding, Wenjian Wang, Qiyue Liu

**Affiliations:** 1 Department of Mechanical and Electrical Engineering, Qingdao University of Technology, Linyi 273400, China; 2 School of Mechanical Engineering, Southwest Jiaotong University, Chengdu 610031, China

**Keywords:** nano-additives, WS_2_, carbon coating, wear, friction, self-repairing

## Abstract

WS_2_-C is produced from a hydrothermal reaction, in which WS_2_ nano-sheets are coated with carbon, using glucose as the carbon source. In order to investigate the tribological properties of WS_2_-C as a lubricant additive, WS_2_-C was modified by surfactant Span80, and friction tests were carried out on an MRS-10A four-ball friction and wear tester. The results show that Span80 can promote the dispersibility of WS_2_-C effectively in base oil. Adding an appropriate concentration of WS_2_-C can improve the anti-wear and anti-friction performance of the base oil. The friction coefficient reached its lowest point upon adding 0.1 wt % WS_2_-C, reduced by 16.7% compared to the base oil. Meanwhile, the wear scar diameter reached its minimum with 0.15 wt % WS_2_, decreasing by 26.45%. Moreover, at this concentration, the depth and width of the groove and the surface roughness on the wear scar achieved their minimum. It is concluded that WS_2_-C dispersed in oil could enter friction pairs to avoid their direct contact, thereby effectively reducing friction and wear. At the same time, WS_2_-C reacts with the friction matrix material to form a protective film, composed of C, Fe_2_O_3_, FeSO_4_, WO_3_, and WS_2_, repairing the worn surface.

## 1. Introduction

For a long time, lubricating oil was the first choice to solve the problem of friction and wear due to its high effectiveness. The advent of nano-additives in lubricant [1,2,3], allowing a better performance in terms of tribological characteristics, greatly improves the lubricity of traditional lubricants.

Due to their great lubricity, carbon material [4,5,6,7] and flake nano-WS_2_ are used as nano-additives in lubrication. Sade et al. [8] demonstrated that nano-WS_2_ modified by Span80 could noticeably reduce the friction coefficient, as well as the depth and width of wear scars, using a ball-on-disc test. Wu et al. [9] compared the friction properties of micro- and nano-flake WS_2_ as lubricant additives. Their results showed that the lubricating performance of WS_2_ with a diameter of 90 nm was slightly better than that of WS_2_ with a diameter of 2 μm, but the difference in friction coefficient was only about 5%. This means that WS_2_ with a diameter of microns also has good lubricating performance. Cao [10] pre-etched the matrix material, and then used a lubricant with WS_2_ particles to carry out ball-milling tests. His results showed that the scratches were repaired, and the main elements in the repaired area were tungsten element (W), sulfur element (S), and oxygen element (O), indicating that the WS_2_ additive had good self-repairing performance. Kumar [11] added graphene to wear-resistant coating, which led to a reduction in the friction coefficient to 0.04, and a significant increase in sliding cycles of the coating. Zheng et al. [12] made a new material named WS_2_/GP by mixing WS_2_ and graphene, and proved that WS_2_/GP had better anti-wear and friction reduction performance than adding either WS_2_ or GP as a lubricant additive. This proved the existence of a synergistic effect between the two nanoparticles.

In this paper, we introduce a new nanomaterial labeled WS_2_-C, prepared by coating carbon on WS_2_ nano-sheets in a hydrothermal reaction. Furthermore, we find some interesting characteristics of this new nanomaterial after specific investigations in tribological tests. Compared to original WS_2_ nano-sheets, this new nanomaterial can reduce the friction coefficient and the diameters of the wear scars when used as an additive in base oil. In particular, we focused on the tribological properties of the particles at different concentrations, and obtained the optimal scope of concentration for reducing friction. Meanwhile, in these experiments, the dispersibility of the nanoparticles in base oil was noted and improved. At last, the micro-morphology and elements of the nanoparticles and wear scars in these experiments were studied to explain the characteristics mentioned before.

Furthermore, it was verified that carbon was coated on the WS_2_ nano-sheets successfully, and this improved their performance in the tribological tests. The dispersibility of the nanoparticles was crucial to the tribological characteristics of the lubricant. Simultaneously, the chemical reaction between the matrix material and WS_2_-C produced a self-repairing film which attached to the surface of friction pair, thereby inhibiting the further wear of the matrix material.

## 2. Materials and Methods

### 2.1. Preparation of Nanoparticles and Lubricants

Firstly, WS_2_-C was prepared by coating carbon material on the surface of WS_2_ nano-sheets under sealed conditions at 180 °C, with glucose as a carbon source. The specific operation was to add 1 g of glucose into 25 mL of deionized water and stir it thoroughly. After the glucose was completely dissolved, 1 g of powdered WS_2_ nano-sheets was added. Then, uniform stirring and ultrasonic treatment were carried out for 30 min. Next, they were put into a hydrothermal autoclave to react for 4 h in a sealed environment at 180 °C. The product, which was named WS_2_-C, was obtained from the autoclave by cleaning and filtering with deionized water and absolute ethanol three times, and drying at 90 °C for 12 h.

Secondly, the surface of dried WS_2_-C nano-sheets was modified to be lipophilic with activator Span80, which can alleviate its agglomeration rate in base oil 500SN. The specific operation steps were as follows: 1 g of WS_2_-C nano-sheets were added to a beaker containing 10 g of Span80, stirring for 3 h at 75 °C with a constant-temperature magnetic stirrer, then filtered and washed with absolute ethanol, and dried in a drying chamber at 90 °C for 4 h. The resulting WS_2_-C powder modified by Span80 was labeled WS_2_-CS. In the same procedure, WS_2_ nano-sheets were modified with Span80 and labeled WS_2_-S to complete the comparative experiment.

**Figure 1:**
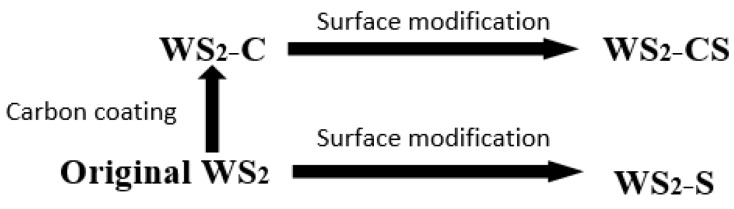
Schematic diagram of the preparation of nanoparticles.

For these complex tags to be easily understood, the preparation progress is shown as a schematic diagram in Figure 1.

In order to study the dispersibility of WS_2_-CS nano-sheets in 500SN base oil, the WS_2_-CS lubricant was prepared by adding 0.1 g of WS_2_-CS nano-additive into 19.9 g of 500SN base oil, stirring evenly with a glass rod. Later, ultrasonic treatment was carried out for 30 min. WS_2_ lubricant, WS_2_-C lubricant, and WS_2_-S lubricant were also prepared according to the above steps. The dispersions of lubricating oil were sealed for five days to check their stability.

### 2.2. Tribological Performance Test

In the test, steel balls, with surface hardness of 64HRC–66HRC and a surface roughness (Ra) of 0.02 μm, were GCr15 secondary standard steel balls with a diameter of 12.7 mm provided by Shanghai Steel Ball Factory (Shanghai, China). These balls were made of bearing steel, whose components and mechanical properties are shown in Table 1 and Table 2. The base oil 500SN was supplied by Guangdong Zhonghai Nanlian Energy Co., Ltd. (Maoming, China). The WS_2_ nano-sheets were produced by Beijing Deco Daojin Science and Technology Co., Ltd. (Beijing, China).

**Figure 2:**
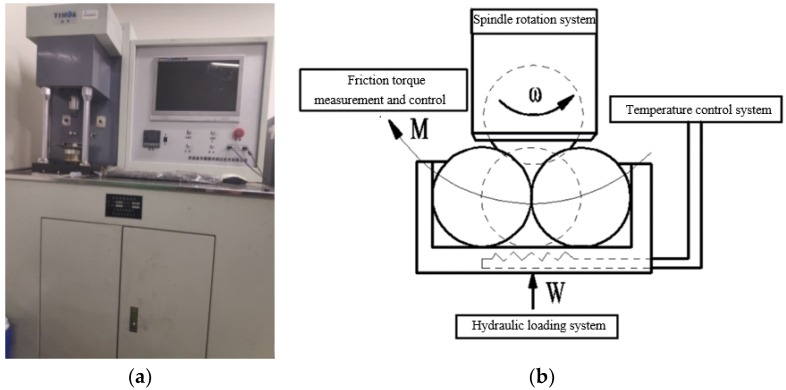
MRS-10A four-ball friction and wear testing machine: (**a**) picture; (**b**) schematic diagram.

The tribological properties of lubricating oil containing WS_2_-C nano-additives were tested on an MRS-10A four-ball friction and wear tester produced by Jinan Yihua Tribological Testing Technology Co., Ltd. (Jinan, China). Pictures and schematic drawings of the test machine are shown in Figure 2. The tester had the function of automatically collecting and storing the friction coefficient in every 10 s. After the test, the friction coefficients were sorted. The tests were carried out under the following conditions: load = 392 N, speed = 600 rpm, temperature = 40 °C, and duration = 60 min. These conditions refer to standard SH-T0762-2005, with minor modifications to the temperature, as discussed later.

In order to study the effect of carbon coating on the lubricating performance of the WS_2_ nano-sheet additives, different types of lubricating oils were prepared as shown in Table 3. To further verify the tribological properties of the WS_2_-C nano-sheets as lubricant additives, lubricants with different concentrations of the additives were prepared as shown in Table 4. The tribological properties of different oil samples were tested through the above experimental parameters.

### 2.3. Characterization of Nanoparticles and Wear Scars

The methods described here were used to characterize the micro-composition of materials. The wear scar diameter was measured with a special optical microscope installed on the tester. Meanwhile, the micro-morphologies of WS_2_-C nano-sheets and wear scars were observed by scanning electron microscopy (SEM, JSM 7800F, JEOL Co., LTD, Tokyo, Japan), while the chemical elements were analyzed by an energy-dispersive spectrometer (EDS). The size and lattice spacing of the WS_2_-C nano-sheets were analyzed by transmission electron microscopy (TEM, JEM-2100F, JEOL Co., LTD, Tokyo, Japan) and high-resolution transmission electron microscopy (HRTEM), and the coating effect of carbon materials on the WS_2_ surface was analyzed by Raman spectroscopy Shamrock SR-500i, Andor Technology plc, Belfast, UK). X-ray photoelectron spectroscopy (XPS, ESCALAB 250Xi, Thermo Fisher Scientific, Waltham, MA, USA) was used to analyze the valence states and chemical compositions of characteristic elements on the surface of wear scars to verify whether the chemical reaction between nano-sheets and matrix materials formed self-repairing protective films. The tests were performed in a vacuum (less than 5 × 10^−10^ mbar). Its survey scan covered a scope of 1400 eV to check the characteristic elements. Survey spectra were obtained with 100 eV of pass energy with a resolution of 1 eV. Additionally, a constant pass energy of 30 eV with a resolution of 0.05 eV was used for more detailed analyses. The data from XPS tests were dealt with using XPS PEAK41 software.

## 3. Results

### 3.1. Characterization of Nanoparticles

**Figure 3:**
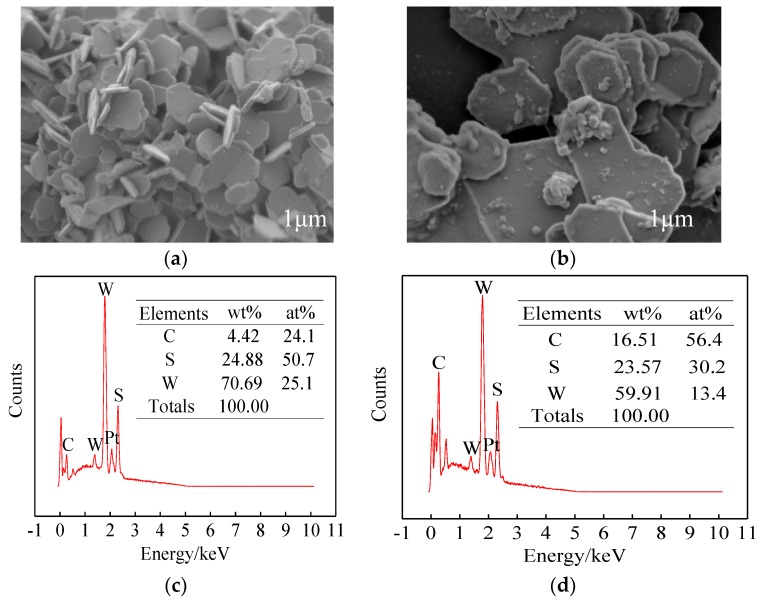
SEM pictures and energy-dispersive spectroscopy (EDS) analyses of (**a**,**c**) WS_2_, and (**b**,**d**) WS_2_-C.

The micro-morphology of the WS_2_ nano-sheets without carbon coating is shown in Figure 3a. The thickness of the WS_2_ nano-sheets was about 50–70 nm, while the width was about 0.2–1 μm. EDS analysis of the WS_2_ nano-sheets, shown in Figure 3c, indicates that the atomic ratio of S and W was about 2.02, which is roughly the same as in WS_2_. The microcosmic morphology of the WS_2_-C nano-sheets with carbon coating is shown in Figure 3b, in which the width of the nano-sheets increased sharply. This planar growth of WS_2_ nano-sheets was related to the high temperature and high pressure in the hydrothermal reaction, while the low growth in thickness was due to the carbon coating. In the EDS element analysis of WS_2_-C nano-sheets (Figure 3d), it was found that the carbon content of WS_2_-C nano-sheets was significantly higher than that of uncoated WS_2_ nano-sheets (Figure 3c), indicating the presence of a carbon layer covering the nano-flake.

**Figure 4:**
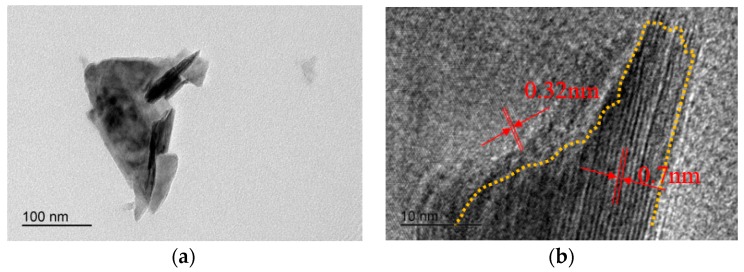
Micro-morphology of WS_2_-C nano-sheets from (**a**) TEM, and (**b**) high-resolution TEM (HRTEM).

**Figure 5:**
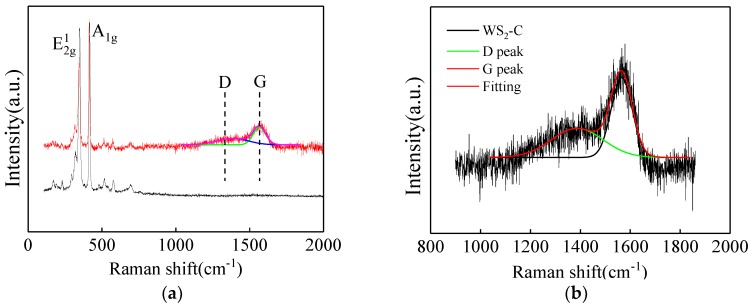
Raman spectra of WS_2_-C: (**a**) full spectra; (**b**) fitting spectra of D and G peaks.

The TEM observation of WS_2_-C nano-sheets is shown in Figure 4. Combined with the observation from SEM, it was found that the width of WS_2_-C nano-sheets was different, ranging from 150 nm to 2 μm. The lattice spacing of WS_2_-C nano-sheets was observed in HRTEM photographs, and it was found that the spacing of crystal planes around 0.32 nm was the same as that of carbon material (002), and the spacing of crystal planes around 0.70 nm was the same as that of WS_2_ material (002). Raman spectra of WS_2_ and WS_2_-C nano-sheets were analyzed as shown in Figure 5a. The spectrum of the latter showed clear D and G peaks, and its Gaussian fitting is shown in Figure 5b. The strength ratio of D and G peaks was about 0.21 for I_D_/I_G_, while it was 1.0 for carbon materials. This was due to the interaction between WS_2_ and carbon atoms on the nano-sheets, which further proved the success of carbon coating [12].

### 3.2. Dispersibility Test of Nanoparticles

**Figure 6:**
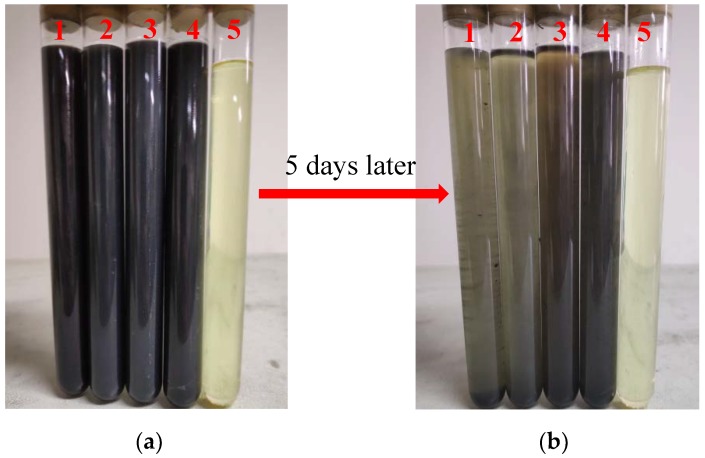
Dispersion of additives (**a**) freshly prepared and (**b**) sealed for 5 days within (**1**) 500SN + 0.5 wt % WS_2_; (**2**) 500SN + 0.5 wt % WS_2_-C; (**3**) 500SN + 0.5 wt % WS_2_-S; (**4**) 500SN + 0.5 wt % WS_2_-CS, and (**5**) pure 500SN.

**Figure 7:**
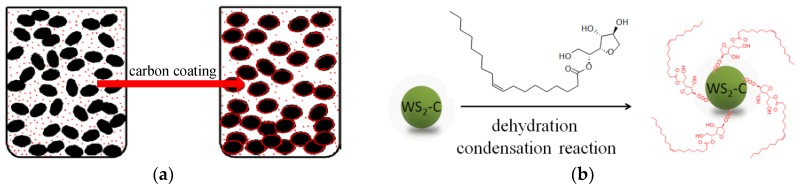
Schematic diagram of surface chemical modification: (**a**) carbon coating; (**b**) surface modification.

The dispersions of lubricating oil sealed five days later were stable. However, the color of the low-concentration lubricant was very light, and it was not easy to check the dispersibility. As shown in Figure 6, the high-concentration lubricants, oils with 0.5 wt % nanoparticles, are displayed. In these pictures, it is clear that the dispersibility of WS_2_-S and WS_2_-CS nano-sheets (3,4) in the base oil was improved after modification with Span80. In particular, the dispersibility of WS_2_-C nano-sheets modified by Span80 (4) was improved most obviously in base oil, while WS_2_ and WS_2_-C nano-sheets without Span80 modification (1,2) completely deposited five days later. This is because the surface of WS_2_-CS nano-sheets was coated with a layer of carbon material, as shown in Figure 7a. The carboxyl (–COOH) which comes from the hydrothermal reaction [13] on the surface of the carbon material reacts with the hydroxyl (–OH) contained in the surfactant Span80 to form a new –COO– bond, which could bond the WS_2_-CS nano-sheets with the molecules of surfactant Span80 at a certain temperature, as shown in Figure 7b. This is the reason why the dispersibility of WS_2_-CS nano-sheets was improved in organic solvent 500SN base oil compared to that of WS_2_ (or WS_2_-S).

### 3.3. Tribological Performance Test

**Figure 8:**
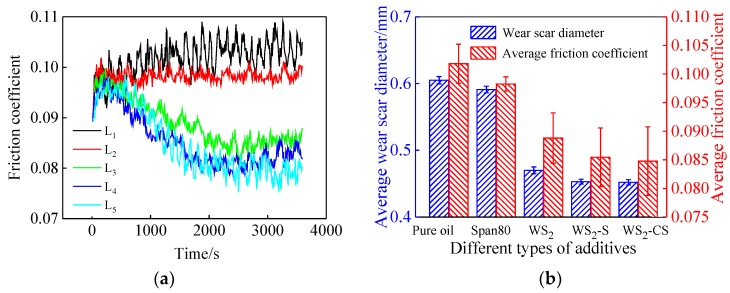
Curves of different additives: (**a**) varying friction coefficient; (**b**) average diameter of wear scar.

**Figure 9:**
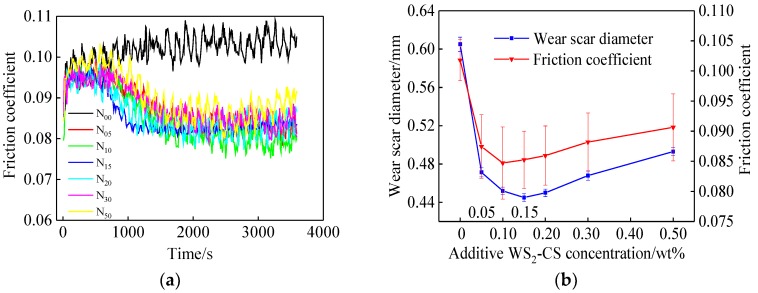
Curves of different additive concentrations: (**a**) varying friction coefficient; (**b**) average diameter of wear scar.

The tribological performance tests were completed under the given conditions with different types of lubricating oils, as shown in the Table 3 and Table 4. Each set of tests was repeated three times, and the average friction coefficients of these data obtained from the tester were calculated. Similarly, the average diameters of the wear scars were determined from three samples of each set. These results are shown in Figure 8 and Figure 9.

It can be seen from Figure 8a that, compared to the pure base oil (L_1_), the friction coefficient decreased only with the addition of the nano-additives (L_3_, L_4_, L_5_), but did not decrease significantly with the addition of activator alone (L_2_). Similarly, in Figure 8b, the averages of friction coefficient and wear spot diameter decreased significantly after adding nanoparticles, and the minimum values were seen for L_5_ (0.1 wt % WS_2_-CS nano-sheets). This indicates that WS_2_ (L_3_, L_4_) and WS_2_-C (L_5_) nano-sheets as lubricant additives can improve the antifriction and antiwear properties of base oil. An initial analysis of the curves of L_4_ and L_5_ suggests that they are roughly the same. However, if the curves are studied in segments, that opinion is abandoned. During the first 1000 s, L_5_ was much higher than L_4_. Gradually, L_5_ started decreasing and approaching L_4_ at 1000 s. Then, at 3000 s, the former surpassed the latter. This means that the lubricant with WS_2_-CS nano-sheets (L_5_) was much more stable and durable than the lubricant with WS_2_-S nano-sheets (L_4_). In another words, the lubricating performance of the WS_2_-CS nano-sheets (L_5_) was better than that of the WS_2_-S (L_4_) nano-sheets at 0.1 wt % concentration. All these data indicate that the carbon coating and the modification with Span80 contributed to the decrease in friction coefficient.

As shown in Figure 9, with the increase in WS_2_-CS nano-sheet concentration, the friction coefficient and wear scar diameter decreased at first, and then increased gradually. The minimum friction coefficient was obtained when the additive concentration was 0.1 wt % (from N_10_), which was 16.79% lower than that of base oil. On the other hand, the minimum diameter of wear scar was from N_15_ (concentration 0.15 wt %), which was 26.45% lower.

**Figure 10:**
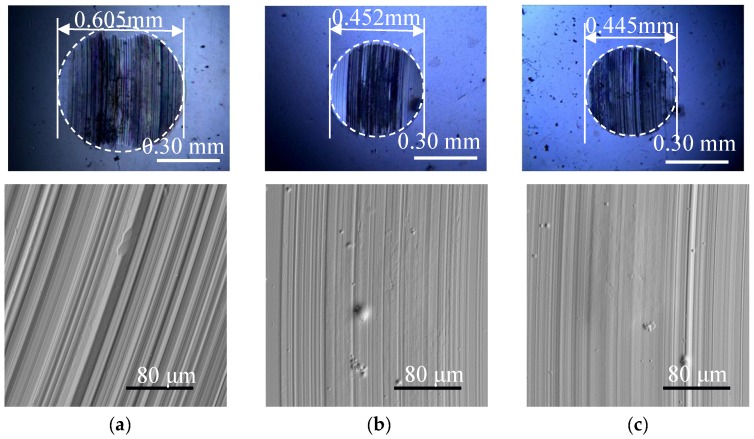

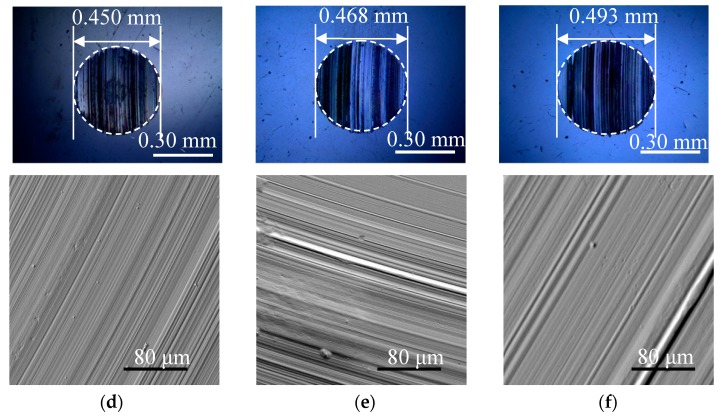
Scanning electron micrographs of abrasion scars from (**a**) pure oil; (**b**) 0.1 wt % WS_2_-CS; (**c**) 0.15 wt % WS_2_-CS; (**d**) 0.2 wt % WS_2_-CS; (**e**) 0.3 wt % WS_2_-CS, and (**f**) 0.5 wt % WS_2_-CS.

Figure 10 shows the surface morphology of the wear scar at different additive concentrations. When using pure base oil 500SN, the wear scar diameter, and the width and depth of the plough groove were obtained at their maximum, and the wear was the most serious. After adding WS_2_-CS nano-additive, the wear scar diameter and surface roughness were obviously reduced. In particular, when the additive concentration was 0.15 wt %, the wear scar diameter was the smallest, and the surface of the wear scar was the smoothest. These results show that the WS_2_-CS nano-additive can improve the surface contact environment of friction pairs and slow down the wear rate. With the increase in additive concentration, the diameters of wear scar, and the depth and width of the plough groove on the surface decreased at first and then increased, which indicates that the additives had an optimal concentration for lubricant use. This phenomenon is related to the agglomeration and chemical curing of nanoparticles with high concentration [14,15]. These agglomerates result in unstable lubrication on the frictional surface [16,17], destroying the lubricating film and weaken the lubricating effect. 

Overall, at the 0.1 wt % concentration, the friction coefficient achieved its minimum and the diameter achieved its second minimum, while, at 0.15 wt % concentration, the diameter achieved its minimum and the friction coefficient achieved its second minimum. Moreover, the surfaces on the wear scars from these two lubricants were much smoother than the others. Therefore, it was concluded that the optimal concentration was between 0.1 wt % and 0.15 wt %.

### 3.4. Elemental Analysis of Wear Scar

**Figure 11:**
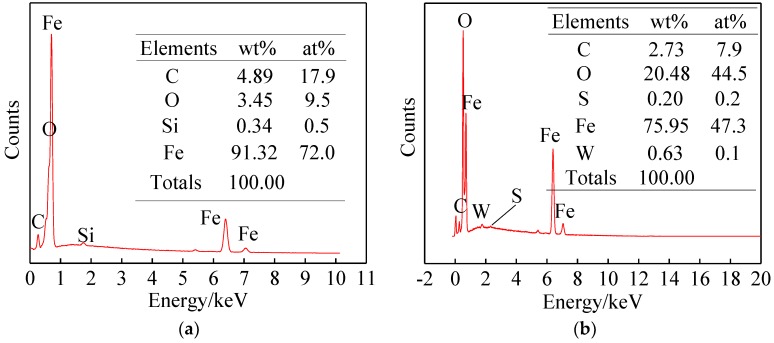
EDS chart on abrasion scars from (**a**) base oil, and (**b**) 0.1 wt % WS_2_-CS lubricant.

**Figure 12:**
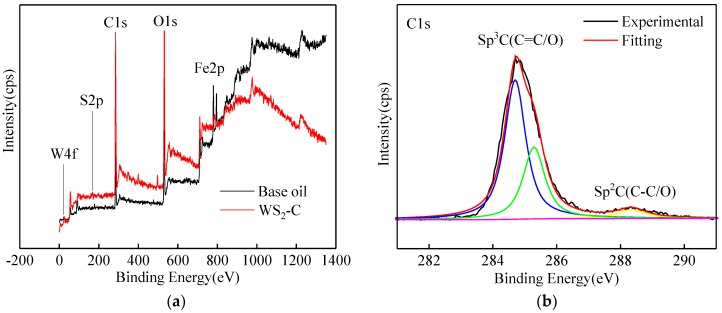

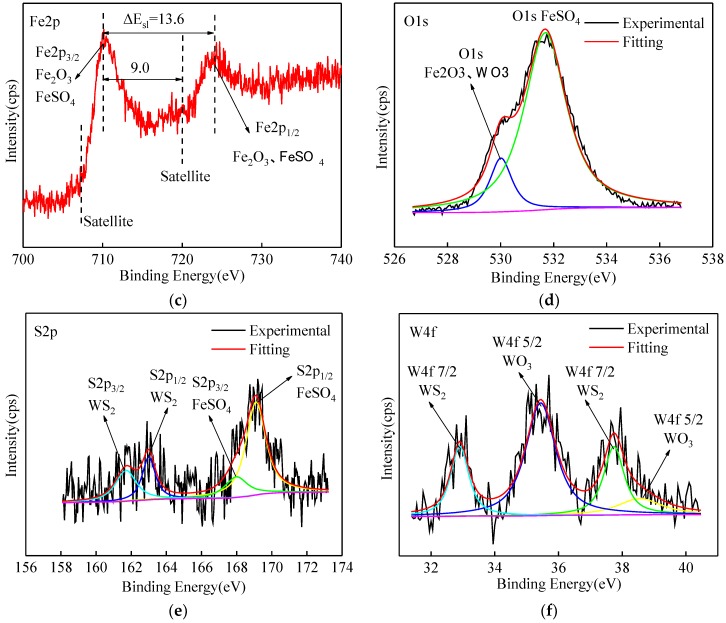
X-ray photoelectron spectroscopy (XPS) spectra from wear scar lubricated with 0.1 wt % WS_2_-C: (**a**) full spectrum; (**b**) C1s; (**c**) Fe2p; (**d**) O1s; (**e**) S2p; (**f**) W4f.

EDS analyses of the wear scar surface using pure base oil and using the lubricant with 0.1 wt % WS_2_-CS were carried out as shown in Figure 11. Some characteristic elements (S and W) were detected from the latter but not the former. XPS analysis was performed to explore the wear scar surface using WS_2_-CS. The presence of characteristic elements (S and W) was found in the full spectrum, as shown in Figure 12a, which is consistent with the EDS results.

Using foreign contaminated carbon (284.8 eV) as a benchmark, the original data were checked, and the peaks of C, O, Fe, S, and W elements were fitted as described below. The peaks at 284.70 eV, 285.29 eV, and 288.35 eV in the C1*s* spectrum represent the bonds of C–C/O and C=C/O. They were derived from external pollution sources and coating carbon materials on the WS_2_ surface. This shows that carbon on the WS_2_-CS surface participated in the friction process and played a role in lubricating the friction pairs [12]. A comprehensive analysis of peak separation spectra of the O, Fe, S, and W elements showed several results. The fitting peaks of O1s, Fe2p, and S2p with peak values at 531.6 eV, 169.10 eV, and 167.98 eV indicated the presence of FeSO_4_ on the surface of the wear scar [18,19]. Furthermore, the O1s fitting peak and Fe2p peak of 530.01 eV indicated is the presence of Fe_2_O_3_ on the surface of the wear scar. Meanwhile, the O1s fitting peak of 530.01 eV and the W4f fitting peaks of 35.44 eV and 38.55 eV indicated that WO_3_ was produced by the oxidation decomposition of WS_2_ on the surface of the wear scar [20,21]. The WO_3_ produced by tribochemical reaction is very compact, which can effectively improve the wear resistance of the friction pairs on the matrix material. The peak value of SW4f fitting for 163.00 eV, 161.70 eV, 32.90 eV, and 37.74 eV showed that WS_2_ substance existed on the surface of the wear scar [22,23]. Thus, it can be explained that WS_2_-CS nano-sheets entering the friction pair can be attached and deposited on the worn surface, and they can react with the matrix material in a complex environment with the friction pair surface to form a self-repairing protective film. The film composed of C, Fe_2_O_3_, FeSO_4_, WO_3_, and WS_2_ could protect the friction pair from further wear. All these data indicate that the carbon coating contributed to wear resistance.

Unfortunately, we did not observe the film in these SEM pictures; however, the substances generated from the reaction were confirmed by the analyses of EDS and XPS. Actually, only a few of particles dispersed in the oil were attached to the surface of the friction pairs and participated in tribochemical reactions, and the thickness of the film ranged from 20–50 nm [24]. Another explanation is that the film is likely to have been intermittent rather than uniform, and it may have only covered a small part of the area rather than the whole scar. 

### 3.5. Analysis of Lubrication Mechanism

**Figure 13:**
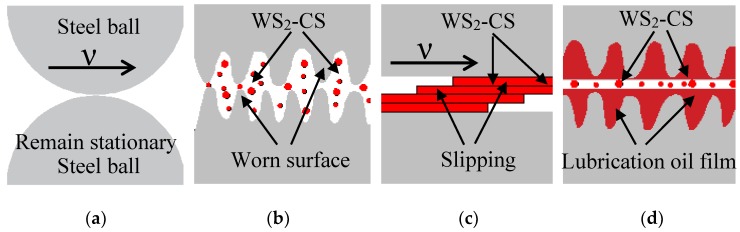
Lubricating mechanism diagram: (**a**) relative movement of the pair; (**b**) distribution and dispersion of the nano-materials; (**c**) interlaminar slide of the nano-sheets; (**d**) formation of the self-repairing film.

Carbon-coated WS_2_ nano-sheets can reduce friction through the good lubricating performance of carbon materials and the slip between WS_2_ layers. During the friction process, WS_2_-CS nano-sheets dispersed in oil enter the friction pair with oil, due to the high-speed relative sliding of the friction pair (Figure 13a). Under the dual action of a large vertical load force between the friction pairs and the high surface energy characteristics of the additive itself, WS_2_-CS nano-sheets are adsorbed and deposited onto the surface of the friction matrix material (Figure 13b). According to its large intergranular spacing, WS_2_ adsorbed on the frictional surface with a lamellar structure is prone to interlaminar slide in the friction matrix (Figure 13c). This reduces the friction force and friction coefficient between the friction pairs. In the complex environment of high temperature and high pressure, WS_2_-CS nano-sheets react with matrix materials to form self-repairing protective films composed of C, Fe_2_O_3_, FeSO_4_, WO_3_, and WS_2_ to further protect the matrix materials of the friction pairs (Figure 13d). At the same time, with the sustained relative motion of the friction pairs, new WS_2_-CS nano-sheets continuously enter the friction pairs to ensure continuous lubrication.

## 4. Discussion

The friction and wear of machine parts are always major reasons of mechanical equipment failure. The employment of nano-additives can effectively improve the physical and chemical properties of pure base oil, and reduce the wear between friction pairs, thereby prolonging the service life of mechanical equipment. 

With the further study of the lubricating mechanism of lubricant additives, it was found that WS_2_ lamellar nano-sheets can reduce the friction coefficient between friction pairs because of the weak interlayer interaction force, resulting in interlayer slip occurring easily. Meanwhile, carbon material is recognized as a material with good lubricity. The ferrite, austenite, and cementite formed by the infiltration of carbon into steel can improve the tribological properties of the material including its strength, hardness, and wear resistance. Therefore, WS_2_-C was prepared via a hydrothermal reaction, in which the WS_2_ nano-sheet as a reactant was coated with carbon, using glucose as the carbon source. The product of the hydrothermal reaction was explored in detail in this paper.

However, nanomaterials are also prone to agglomeration due to their high surface energy. This agglomeration is an important factor affecting the lubricating performance, and it is difficult to exploit the properties of nanomaterials. From the literature, it was found that surfactant Span80 has a good effect on modifying nanoparticles to alleviate agglomeration [12], as does WS_2_-C, verified in this study. 

In the tests carried out, the conditions reflect standard SH-T0762-2005, other than a slight modification in temperature. The temperature at 40 °C simulated the ideal environment at room temperature. Compared to 75 °C in the standard, the decrease in temperature increases the viscosity of the lubricating oil, and increases the thickness of the oil film in the friction pairs, thereby reducing the friction coefficient. The temperature factor is important for the friction test, as are factors of load and speed and concentration. While this paper focused on concentration, our research also looks at the effects of different speeds, loads, and temperatures. Studies on other factors are ongoing at the moment.

In addition, the concentration of additives has great influence on the tribological properties of the lubricating oil. Excellent lubricating performance is not achieved by increasing the concentration of nano-additives in a linear fashion. The proper concentration of additives is a prerequisite for obtaining better lubricating performance. In this paper, the optimal concentration of nano-WS_2_-C for friction reduction and wear resistance was obtained through experiments. The results showed that the WS_2_-CS material prepared in this study can effectively reduce friction and wear in the base oil 500SN. Compared with the pure WS_2_ additive, its lubricating performance was better.

It should be noted that all the above tests and results were based on this particular environment, including the bearing steel friction pair and 500SN base oil, as well as the nano-additive WS_2_. We need further research to verify whether these conclusions are applicable to other conditions.

In summary, nano-materials as additives can improve the tribological properties of lubricating oil, and nano-WS_2_ coated by carbon has better characteristics than ordinary WS_2_. Thus, it may be a new path to further promoting the effect of other nanoparticles that show good tribological characteristics as lubricants. 

## 5. Conclusions

We prepared new nanoparticles named WS_2_-C, which featured coated carbon on the surface of WS_2_ nanosheets in a hydrothermal reaction. To improve the stability and durability of lubricants, we modified them using activator Span80. After that, lubricants using nanoparticles of different concentrations were investigated on the MRS-10A four-ball friction and wear tester. The data from these experiments were analyzed and discussed through the use of SEM, TEM, HRTEM, EDS, XPS, etc. Finally, we came to the following conclusions:

- Carbon materials were successfully coated on the WS_2_ nanosheets via hydrothermal reaction using glucose as a carbon source. The dispersibility of WS_2_-C nano-sheets modified by Span80 was significantly improved in base oil.
- The friction coefficient of WS_2_-CS nano-sheets achieved its minimum at the concentration of 0.1 wt %, which was 16.79% lower than that of base oil, and the diameter of wear scar achieved its minimum at the concentration 0.15 wt %, which was 26.45% lower than that of base oil. The optimal scope of concentration for reducing friction ranged from 0.1 wt % to 0.15 wt %.
- Interlayer slip occurred on the WS_2_-CS nano-sheets when oil entered the friction pairs. At the same time, the friction force was reduced, and the chemical reaction with the matrix material produced a self-repairing film composed of C, Fe_2_O_3_, FeSO_4_, WO_3_, and WS_2_, which inhibited the further wear of the matrix material.

## Figures and Tables

**Table 1:** Composition of bearing steel.

Element	C	Mn	Si	S	P	Cr	Ni
Content	0.95	0.30	0.25	0.02	0.02	1.5	0.20

**Table 2:** Mechanical properties of GCr15 bearing steel.

Material	Heat Treatment Method	σ_s_ (MPa)	σ_b_ (MPa)	E (GPa)	Hardness (HRC)
GCr15 bearing steel	Oil quenching and low-temperature tempering	1700–1814	2157–2550	250	64–66

**Table 3:** Lubricating oils with different types of additives.

Lubricant Number	Composition of Lubricating Oil
L_1_	Pure base oil 500SN
L_2_	500SN + Span80 (0.1 wt %)
L_3_	500SN + WS_2_ (0.1 wt %)
L_4_	500SN + WS_2_-S (0.1 wt %)
L_5_	500SN + WS_2_-CS (0.1 wt %)

**Table 4:** Lubricating oils with different additive concentrations.

Lubricant Number	Composition of Lubricating Oil
N_00_	Pure base oil 500SN
N_05_	500SN + WS_2_-CS (0.05 wt %)
N_10_	500SN + WS_2_-CS (0.1 wt %)
N_15_	500SN + WS_2_-CS (0.15 wt %)
N_20_	500SN + WS_2_-CS (0.2 wt %)
N_30_	500SN + WS_2_-CS (0.3 wt %)
N_50_	500SN + WS_2_-CS (0.5 wt %)
